# Perceived stress is related to lower blood pressure in a Swedish cohort

**DOI:** 10.1177/14034948211030352

**Published:** 2021-07-20

**Authors:** Maud Miguet, Gaia Olivo, Diana-Maria Ciuculete, Sölve Elmståhl, Lars Lind, Helgi B. Schiöth

**Affiliations:** 1 Department of Neuroscience, Functional Pharmacology, Uppsala University, Sweden; 2 Skånes Universitetssjukhus (SUS), Sweden; 3 Uppsala Clinical Research Center (UCR), Sweden; 4 Department of Medical Sciences, Uppsala University, Sweden; 5 Institute for Translational Medicine and Biotechnology, Sechenov First Moscow State Medical University, Russia

**Keywords:** Stress, job strain, blood pressure, hypertension

## Abstract

**Aims:**

General psychosocial stress and job strain have been related to blood pressure (BP) with conflicting results. This study sought to explore the contribution of several lifestyle factors in the relation between general psychosocial stress, job strain and BP.

**Methods:**

This cross-sectional study investigated the association of general stress and job strain with systolic BP (SBP) and diastolic BP in a sample of 9441 employed individuals from the EpiHealth cohort. General stress was measured by the Perceived Stress Scale. Job strain was assessed with the Job Content Questionnaire, assessing two dimensions of job strain: psychological job demand and decision latitude. Linear regression and sensitivity analysis were performed.

**Results:**

At the uncorrected model, general stress, job demand and decision latitude were all inversely associated with SBP. After further adjustment for lifestyle and health parameters, only general stress was associated with SPB (β coefficient: −0.103; 95% confidence interval −0.182 to 0.023).

**Conclusions:**

**General stress is associated with lower SBP independently of lifestyle in middle-aged adults. Our findings point towards a major contribution for job-unrelated stressors in determining SBP and support the pivotal role of lifestyle behaviours and health status in modulating the effect of stress on BP, calling for a careful selection of confounders.**

## Introduction

Psychological stress has long been known to be a risk factor for cardiovascular diseases (CVD) [1,2] due to its neuroendocrine hypertensive effects [1]. Hypertension is considered as one of the most important risk factors for cardiovascular disease (CVD) [3]. However, the relationship between chronic psychological stress and CVD is remarkably complex due to its intertwining with other lifestyle risk factors such as high body mass index (BMI) [4], smoking and lower physical activity [5]. In fact, chronic psychological stress can lead to unhealthy lifestyle behaviours [1].

Work-related stress has been reported to be a major contributor to high blood pressure (BP) [1,2], though findings regarding the association of job strain with BP and CVD are still conflicting [6]. Some studies have found positive associations between work-related stress and hypertension, while others have not found any association or have even reported negative associations. Many factors can impact this association. First, when taking the above-mentioned lifestyle factors into account, the impact of job strain on systolic BP (SBP) seems to decrease [7]. The use of antihypertensive medication [8] or the type of occupation [3] should also be considered. Moreover, the impact of job strain on BP seems to be dependent on sex [9–11], having been more consistently observed in men rather than women [7,9,10]. In men, lifestyle and job strain, as measured by the Job Content Questionnaire (JCQ), were found to be as reliable as traditional clinical risk factors in predicting CVD in a 14-year prospective study [12]. Nonetheless, the impact of psychosocial features of the working environment on women’s cardiovascular health is not to be underestimated [13], as it can be amplified by other social factors such as lack of husband support and high burden in family duties [9].

**Figure 1.**
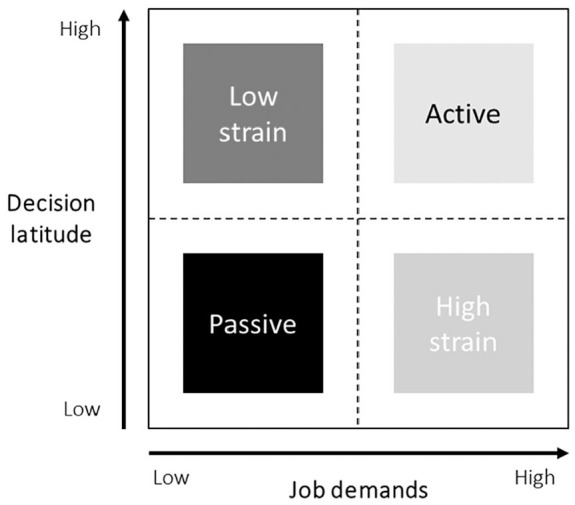
Job strain model by Karasek [14].

Interestingly, different results have been reported in relation to SBP and diastolic BP (DBP), with a mean difference in SBP and DBP between stressed and not stressed groups ranging from +2 to +10.2 mmHg and +2 to +17.97 mmHg [9], respectively. Plus, in the same study, odds ratios for hypertension among stressed groups ranged from 1.18 to 2.9 [9]. Moreover, some studies have pointed towards the importance of considering the two dimensions underlying job strain as separate contributors to BP [1]. According to the Karasek–Theorell model [14], job strain stems from the combination of high job demands and low decision latitude [14] (Figure 1). Job demand can be defined as the need to work quickly and hard, while decision latitude reflects the lack of control over skill use, time allocation and organisational decisions [15]. High demand and low decision latitude have largely been associated with higher risk for CVD [6], and low job control in particular has been reported to be associated with elevated DBP in men [1].

### Aims

The role of social and job-unrelated stress is less documented, and there is no consensus over its effects on BP [16]. The discrepancies between results of studies might be partly due to the heterogeneity of the samples recruited and partly due to the confounding effect of lifestyle and individual personality [17]. In this study, we investigated the association of SBP and DBP with general stress and job strain, as measured by the Perceived Stress Scale (PSS) [18] and the JCQ [19], respectively, using data from the EpiHealth cohort. The large number of participants (*N*=9441) allowed us to explore the contribution of several lifestyle factors, such as alcohol consumption, smoking, sleep, BMI, physical activity and health status.

## Methods

### Study population

The EpiHealth is a longitudinal study based on the Swedish population [20]. The study consists of a collection of data on lifestyle factors by self-assessment using an Internet-based questionnaire, followed by a visit to a test centre where blood samples are collected and physiological parameters are recorded. Please refer to Lind et al. [20] and to the EpiHealth website (https://www.epihealth.se/) for detailed information regarding the recruitment and study design. Briefly, the EpiHealth study started in 2011. Participants were randomly selected and invited between 2011 and 2017 from the population registry in the Swedish cities of Malmö and Uppsala by analogue mail. The participation rate was about 20%. The EpiHealth study has been approved by the Ethics Committee of Uppsala University at the start of the pilot phase of the study and before the continuation of the study (Log. No. 2010/402) and by the Swedish Data Inspection Board (No. 307-2011). The EpiHealth study ethical policy conforms to the Declaration of Helsinki and complies with the Swedish Personal Data Act, Genetic Integrity Act and the Biobank Act.

At the time of data collection, the cohort consisted of 23,679 individuals, with a mean age of 60.3 years (range 45–75 years). In the present study, we included those participants who reported being properly employed in response to the question ‘Which of the following options best describes your current situation?’. The different response options were: ‘Employee’, ‘Unemployed’, ‘Runs own company or part-owned company’, ‘Retirement pension’, ‘Activity or sickness compensation (early retirement pension) due to illness/disability’, ‘Sick leave (for two months or longer)’, ‘Parental leave (for two months or longer)’, ‘Student’, ‘On leave’, ‘Housewife/husband’ or ‘Other and do not know/do not want to answer’. A total of 11,044 individuals were employed and were eligible for inclusion in this study. Individuals with a SBP ⩾180 mmHg or a DBP ⩾120 mmHg (hypertensive crisis) [21] as well as those taking antihypertensive medication were excluded, leaving a final sample of 9441 participants.

### Primary study variables

Our primary outcomes were SBP and DBP. These were measured at the EpiHealth assessment centres located in Malmö and Uppsala, Sweden. First, a simple one-lead electrocardiogram (ECG; lead I) was taken for 30 seconds by contact between the participant’s hands and ECG electrodes (Zenicor-EKG^®^; Zenicor, Stockholm, Sweden) to screen for atrial fibrillation and other arrhythmias [20]. BP and pulse rate were then recorded twice in the sitting position by an automatic device (Omron, Kyoto, Japan). If BP was >180/110 mmHg or if a previously unknown atrial fibrillation was detected, the participant was recommended an urgent check-up by their general practitioner. For rates of atrial fibrillation of >100 bpm, the participant was referred to the nearest emergency department.

General stress was measured with the Swedish version [22] of the 10-item PSS (PSS-10) questionnaire [18]. This questionnaire assesses how unpredictable, uncontrollable and overloaded the individuals rate their lives in general. It consists of 10 questions about how often the participants feel a certain way: ‘In the last month, (1) How often have you been upset because of something that happened unexpectedly? (2) How often have you felt that you were unable to control the important things in your life? (3) How often have you felt nervous and “stressed”? (4) How often have you felt confident about your ability to handle your personal problems? (5) How often have you felt that things were going your way? (6) How often have you found that you could not cope with all the things that you had to do? (7) How often have you been able to control irritations in your life? (8) How often have you felt that you were on top of things? (9) How often have you been angered because of things that were outside of your control? (10) How often have you felt difficulties were piling up so high that you could not overcome them?’. Responses ranged between 0=‘never’ and 4=‘very often’. Questions 4, 5, 7 and 8 were reverse scored. The total score can range from 0 to 40, with higher scores indicating higher perceived stress.

Job strain was assessed with the Swedish version of the JCQ [23]. This is an 11-item questionnaire assessing two dimensions of job strain: psychological job demand and decision latitude. Psychological job demand was measured by five items: velocity, difficulty, effort, time (reverse scored) and conflict. Decision latitude was assessed by six items: learning, skill, creativity, repetitiveness (reverse scored), decision and responsibility items. Responses to each item on job demand and decision latitude was scored on a four-point ordinal scale where 1=‘yes, often’, 2=‘yes, rather often’, 3=‘no, seldom’ and 4=‘no, never’. The score sums were calculated for each index of questions about job demand and decision latitude. The scale of job demand ranged from 5 to 20 and was dichotomised by median score into low demand (a score of 5–11) and high demand (a score of 12–20). The scale of decision latitude ranged from 6 to 24 and was dichotomised by median score into low decision latitude (a score of 6–19) and high decision latitude (a score of 20–24). According to the job strain model (Figure 1), the index combined the dichotomised variables into four different types of work situations: low strain jobs (low demand, high control), high strain jobs (high demand, low control), passive jobs (low demand, low control) and active jobs (high demand, high control).

### Confounding variables: demographics, health and lifestyle factors

After reporting demographics covariates (sex, age, civil status and education level), the participants were asked to report other information regarding lifestyle behaviour, health status and occupation, since these factors have all been associated with BP [24]. Alcohol intake frequency was reported as: ‘never’, ‘once a month or less’, ‘2–3 times a month’, ‘once a week’, ‘2–3 times a week’, or ‘4 times a week or more often’. Leisure physical activity level was recorded on a seven-point scale, with examples of what type of activity belonged to each level. Sleep duration was reported on a seven-point scale from ‘less than four hours’ to ‘more than 10 hours’. Smoking status was a binary response and recorded as ‘current smoker’: ‘yes’ or ‘no’. Height was measured, and weight was recorded on a scale using bioimpedance to calculate fat mass (Tanita, Tokyo, Japan) [23]. BMI and body fat percentage were recorded. Waist-to-hip ratio (WHR) was also measured. As WHR is considered a better indicator of all-cause mortality compared to BMI, we chose to use WHR instead of BMI for statistical analyses [25].

Information was also recorded regarding current job. Length of working week was reported from the following options: ‘⩽5 hours’, ‘6–10 hours’, ‘11–20 hours’, ‘21–30 hours’, ‘31–40 hours’, ‘41–50 hours’ or ‘>50 hours’. Participants were also asked to report whether they did shift-work, had done shift-work before or never did shift-work.

Finally, information was recorded regarding current diseases, namely chronic obstructive pulmonary disease, asthma, diabetes, heart failure, stroke, heart attack, atrial fibrillation and depression. The use of a pacemaker was also noted. Finally, the use of medication for diabetes or to lower cholesterol was recorded.

### Statistical analyses

Statistical analyses were carried out with IBM SPSS Statistics for Windows v24 (IBM Corp., Armonk, NY). Descriptive statistics of our primary study variables are reported in Table I. Continuous variables are presented as the mean±standard deviation, and categorical variables are presented as percentage and number.

**Table I.** Characteristics of study participants.

a. Continuous variables	Missing	***M* (*SD*)**
PSS	4.8%	12.8 (5.7)
Job demand	3.4%	12.3 (3.0)
Decision latitude	1.3%	19.6 (2.5)
SBP (mmHg)	None	128.4 (14.9)
DBP (mmHg)	None	80.8 (9.3)
Age (years)	1.1%	54.2 (6.0)
BMI (kg/m^2^)	1.1%	25.7 (3.8)
WHR	<0.1%	0.9 (0.1)
Body fat percentage	1.1%	30.1 (8.1)
b. Categorical variables	Missing	Valid frequency
Sex (females)	None	62.6%
Civil status (married)	0.1%	57.0%
Education (university)	0.1%	55.2%
Shift-work (yes, currently)	0.4%	10.2%
Sleep hours (6–8 hours)	0.5%	90.6%
Working week (31–40 hours)	0.3%	53.2%
Alcohol intake (2–3 times a week)	2.1%	33.0%
Physical activity (2–4 hours per week of light intensity)	1.0%	30.8%
Smoking status (yes)	54.9%	17.0%
Snus (yes)	85.7%	49.4%
Asthma	0.3%	6.5%
COPD	0.3%	0.8%
Diabetes	0.3%	0.1%
Heart failure	0.3%	0.1%
Heart attack	0.3%	0.3%
Stroke	0.3%	0.4%
Depression	0.4%	11.2%
Pacemaker	None	0.9%
Atrial fibrillation	None	0.4%
Medication for diabetes	None	0.7%
Medication for high cholesterol	None	2.6%

Valid frequency: percent occurrence among the valid (=non-missing) observations.

PSS: Perceived Stress Scale; SBP: systolic blood pressure; DBP: diastolic blood pressure; BMI: body mass index; WHR: waist-to-hip ratio; COPD: chronic obstructive pulmonary disorder.

In order to reduce the number of covariates, we performed a series of analyses of variance to explore the relationship between each covariate and SBP or DBP. Covariates with a significant association (*p*<0.05) were retained for each outcome (Table II).

**Table II.** Covariates of blood pressure.

Outcome measure	Adjusted model	Sensitivity model
SBP	Age Sex Civil status Education Alcohol intake Physical activity Working hours Sleep hours WHR Body fat percentage Depression Medications for high cholesterol	Adjusted model + smoking
DBP	Age Sex Civil status Education Physical activity Sleep hours WHR Body fat percentage	Adjusted model+smoking

We performed separate linear regressions to test the association between SBP and DBP with general stress and job strain scores (both job demand and decision latitude separately) at an uncorrected model. We then performed a sensitivity analysis by running a corrected model, including the covariates found to be significantly associated with the respective outcome variable at the univariate analyses. The threshold for significance was set at *p*<0.025 to correct for multiple testing according to Bonferroni (two independent variables tested).

## Results

### Descriptive statistics

The final sample consisted of 9441 individuals. The percentage of missing data on the covariates was overall small, ranging between 0.1% and 2.1%, except for smoking-related variables. PSS, job demand and decision latitude had 4.8%, 3.4% and 1.3% of missing data, respectively. Descriptive and percentages of missing data are reported in Table I.

The outcome variables were differently associated with the covariates (Table II). Given the high percentage of missing data on smoking status, we included it in an additional sensitivity analysis rather than in the main model.

### SBP

**Figure 2.**
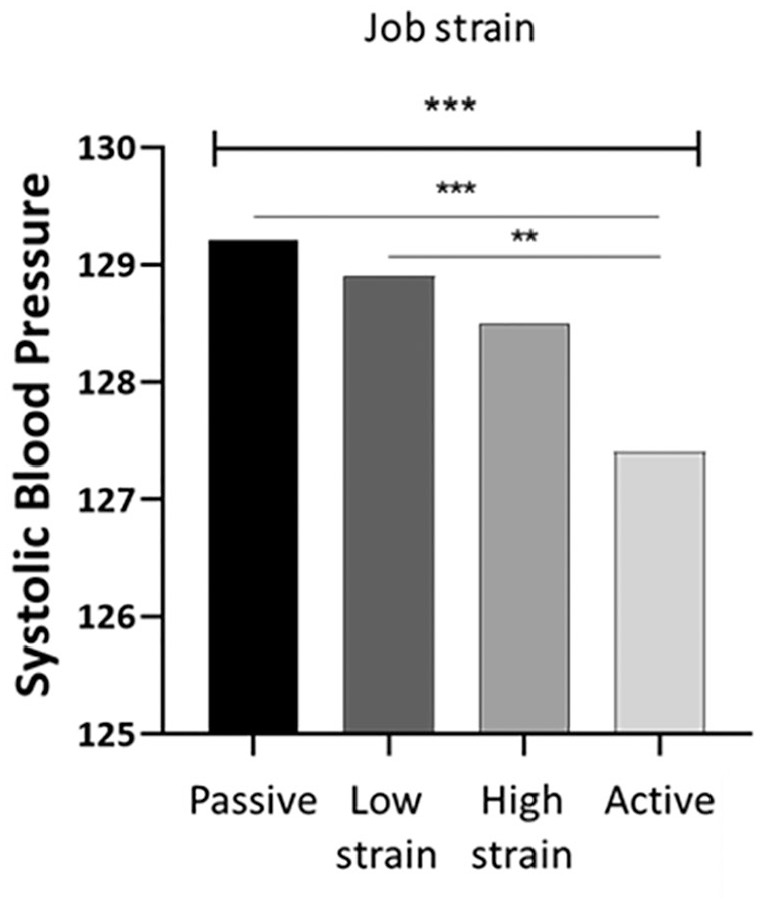
Systolic blood pressure according to job strain categories. Unadjusted comparison (analyses of variance) between job strain groups, Bonferroni post hoc tests. ***p>0.001; **p>0.005.

The three independent variables ‘general stress’, ‘job demand’ and ‘decision latitude’ were inversely associated with SBP at the uncorrected model (Table III). General stress was still significantly inversely associated with SBP when correcting for covariates (β coefficient: −0.104; 95% confidence interval (CI) −0.157 to −0.050; *p*<0.001), and after further correction for smoking (β coefficient: −0.103; 95% CI −0.182 to −0.023; *p*=0.011). However, neither job demand nor decision latitude was associated with SBP after correction for covariates. When considering the independent variable as categories, there were significant differences in SBP between the job strain categories (*p*=0.00005). Lower values of SBP were found in the active job group (Figure 2).

**Table III.** Association between blood pressure and stress.

	SBP β coefficient [95% CI]	*p*	DBP β coefficient [95% CI]	*p*
*General stress*				
Uncorrected (*n*=8961)	−0.172 [−0.225 to −0.118]	4.54E–10*	−0.030 [−0.064 to 0.003]	0.077
Adjusted model (*n*=8598)	−0.104 [−0.157 to −0.050]	0.0001*	−0.023 [−0.056 to 0.010]	0.181
Sensitivity analysis (*n*=3958)	−0.103 [−0.182 to −0.023]	0.011*	−0.001 [−0.050 to 0.048]	0.971
*Job demand*				
Uncorrected (*n*=9095)	−0.259 [−0.362 to −0.156]	8.58E–7*	−0.084 [−0.148 to −0.020]	0.010*
Adjusted model (*n*=8731)	−0.050 [−0.153 to 0.054]	0.347	−0.021 [−0.084 to 0.041]	0.499
Sensitivity analysis (*n*=4010)	−0.060 [−0.213 to 0.093]	0.441	−0.020 [−0.114 to 0.074]	0.678
*Decision latitude*				
Uncorrected (*n*=9292)	−0.146 [−0.269 to −0.022]	0.020*	−0.029 [−0.105 to 0.048]	0.461
Adjusted model (*n*=8905)	−0.049 [−0.176 to 0.079]	0.456	0.019 [−0.059 to 0.097]	0.629
Sensitivity analysis (*n*=4072)	−0.024 [−0.163 to 0.211]	0.805	0.126 [0.011 to 0.240]	0.031

Adjusted model for SBP: age, sex, civil status, education, alcohol intake, physical activity, working hours, sleep hours, WHR, body fat percentage, depression, medication for high cholesterol. Adjusted model for DBP: age, sex, civil status, education, physical activity, sleep hours, WHR, body fat percentage. Sensitivity analyses: adjusted model+smoking. Linear regression coefficients (β) and 95% confidence interval (CI).

* Surviving for multiple testing correction (*p*<0.025).

### DBP

Job demand was inversely correlated with DBP at the uncorrected model (*p*=0.010; Table III). No associations were found at the corrected model between DBP and general stress or job strain (neither job demand nor decision latitude), also when further correcting for smoking status (Table III). When considering the independent variable as categories, there were no differences between the job strain categories (*p*=0.153).

## Discussion

We investigated the association between general stress and job strain on SBP and DBP in a sample of 9441 employed individuals. General stress was measured by the PSS, while job strain was measured using the JCQ, assessing two dimensions of job strain: psychological job demand and decision latitude.

Our sample showed a decrease in SBP with increasing levels of general stress. This is in accordance with previous literature findings. In fact, if psychosocial stress has been reported to be linked to high BP in some studies [16], additional investigations have reported a negative association between stress and BP when confounders are taken into account [25,26]. Several explanations for this paradoxical relationship between high general stress perception and low BP have been proposed [27]. Some theories suggest that in order to cope with adverse circumstances, certain individuals suppress their emotional reaction to preserve their psychological well-being. Continuous exposure to a stressor may lead to habituation and adaptation or even ‘stress denial’. Accordingly, Hildrum et al. reported in a 22-year follow-up Norwegian cohort that anxiety and depression lower BP [28]. Another interpretation supports that the elevation in BP may lead to changes in the perception of negative affects through physiological effects on central nervous system functioning. Indeed, recent studies have reported that elevation in BP progressively reduces sensitivity to acute pain [29]. In terms of this theory, increased BP is a physiological reinforcing mechanism that reduces pain and stress perception as a coping strategy.

Most of the studies reporting a positive association between stress and high BP focused on job-related stress [16] rather than general psychosocial stress. We found higher values of SBP among the passive job group. This is in accordance with the literature, highlighting that a passive condition means a perceived underutilisation of workers and possibly a more stressful or perceived more stressful job, with negative health consequences [13]. However, we did not find any association between job strain and BP when further adjustments were made for covariates. Several factors might have contributed to the lack of detectable associations. First, the length of exposure to psychological work stress might play a role [10]. Furthermore, 55% of our population sample had a university degree, which might suggest that the majority are in a privileged social and job situation that protects them from job stress. Lastly, when considering lifestyle factors, the impact of job strain on BP seems to be reduced [30]. The hypothesis that lifestyle factors might modulate the effect of job strain on BP [7] is supported by our findings, showing a decrease in SBP in the uncorrected model in response to job strain, but not when lifestyle-related and clinical factors were accounted for.

On the other hand, no associations of job strain or general stress with DBP were detected when adjusting for covariates, partially in contrast with previous studies [1]. It is worth noting, however, that the association between job strain and DBP has been reported to be stronger in conditions of mild hypertension rather than normal BP [1], while the mean DBP lays in the lower part of the normal pressure range in our sample.

Our study was based on a Swedish cohort. Thus, our findings are not necessarily reflective of the general population. Moreover, we could not explore the potential role of partner support or the mediatory effect of different personality traits on stress perception. It must also be noticed that BP measurements at work have been proposed as being a more accurate way of measuring the effect of job strain on BP compared to ambulatory measurements [1]. Finally, our study had a cross-sectional design. Thus, no causal mechanisms could be inferred. Future longitudinal studies will have to elucidate further the complex relationship between different aspects of stress and BP.

## Conclusions

We investigated the association between general stress and job strain on SBP and DBP in a sample of 9441 employed individuals. We could not detect an effect of job strain on BP when correcting for lifestyle and health factors, supporting the hypothesis that lifestyle might partially be responsible for the association between high job strain and elevated SBP. On the other hand, general perceived psychosocial stress was associated with a decrease in SBP but not DBP independently of lifestyle. Our findings point towards a major contribution for job-unrelated stressors in determining SBP in the middle-aged population, and they support the notion of a pivotal role for lifestyle and disease state in modulating the effect of stress on BP, calling for careful selection of confounders.
